# The Effects of PROsyntax in Children with Developmental Language Disorder and Autism Spectrum Disorder: A Nonrandomized Controlled Trial

**DOI:** 10.1177/23969415251350586

**Published:** 2025-06-17

**Authors:** Mafalda Azevedo, Alexandrina Martins, Tatiana Pereira, Pedro S. Couto, Marisa Lousada

**Affiliations:** 1 637117 RISE-Health—Department of Education and Psychology, Campus Universitário de Santiago, 386350 University of Aveiro, Aveiro, Portugal; 2 School of Health Sciences, 56062 University of Aveiro, Aveiro, Portugal; 449975 Center of Linguistics of the University of Lisbon, University of Lisbon, Lisbon, Portugal; 3 Center of Linguistics of the University of Lisbon, University of Lisbon, Lisbon, Portugal; 4 Center for Research and Development in Mathematics and Applications, University of Aveiro, Aveiro, Portugal; 5 Department of Mathematics, University of Aveiro, Aveiro, Portugal

**Keywords:** Syntax, intervention, developmental language Disorder, autism spectrum disorder

## Abstract

**Background:**

Children with autism spectrum disorder (ASD) and children with development language disorder (DLD) often experience syntactic impairments. It is of the utmost importance to implement evidence-based intervention at the earliest possible stage to mitigate the adverse effects of these difficulties. Internationally, several programs are supported by scientific evidence. In Portugal, there are currently only two intervention programs, one of which is PROsyntax. However, its effectiveness has not yet been established.

**Aim:**

This study aims to determine the effects of PROsyntax on expressive and receptive syntax in preschool-age children with syntactic impairments diagnosed with DLD or ASD.

**Methods and procedures:**

This study is a nonrandomized controlled trial with a nonprobabilistic convenience sample. Thirty-one children aged between 3 and 6 years were recruited and allocated into an experimental group (EG, intervention group) (*n* = 14) and a control group (CG, without intervention) (*n* = 17). A blind pre- (T1) and postintervention (T2) assessment was conducted using two standardized instruments (SIN:TACS for expression and Subtest 3 of Avaliação da Linguagem Oral (ALO) for comprehension). Children in the EG received intervention with PROsyntax, comprising 24 sessions, biweekly, lasting 1 hr each. The intervention was conducted within the school setting by a speech and language therapist.

**Outcomes and results:**

Statistically significant improvements were observed in the EG compared to the CG in both expressive (F_Time × Group_(1,27) = 293.22; *p* < .001; η_p_^2^ = 0.92) and receptive (F_Time × Group_(1,27) = 147.18; *p* < .001; η_p_^2^ = 0.85) syntax. Large effect sizes were found (SIN:TACS: d = 4.07 (DLD) and d = 11.67 (ASD); ALO: d = 3.29 (DLD) and d = 4.31 (ASD)). Strong correlations between measures were observed at both time points. Postintervention, the CG also received the intervention and showed comparable gains. High satisfaction ratings were reported by both families and early childhood educators.

**Conclusions and implications:**

The findings provide preliminary evidence supporting the effects of PROsyntax in improving expressive and receptive syntactic skills in preschool-age children with ASD or DLD. These findings have important implications for clinical practice, suggesting that explicit interventions can yield significant gains in preschool-age children with syntactic impairment. However, the nonrandomized design, small sample size, and absence of long-term follow-up limit the generalizability of results. Further research is needed to confirm these effects and explore differential responses across diagnostic groups.

## Introduction

Developmental language disorder (DLD) is a heterogeneous neurodevelopmental disorder characterized by difficulties in language acquisition, despite the absence of known biomedical conditions (e.g., brain injury, neurodegenerative diseases, cerebral palsy, or other difficulties related to genetic or neurological causes; Bishop et al., 2017; Sansavini et al., 2021). The condition historically known as specific language impairment has now been widely recognized as DLD following the CATALISE consensus. This consensus emphasized the disorder's developmental nature and its broad impact on language functioning (Bishop et al., 2017). Affecting approximately 7% of children, DLD encompasses challenges across various language domains, including phonology, morphology, syntax, semantics, and pragmatics, affecting production and/or comprehension (Bishop et al., 2017; Norbury et al., 2016). DLD emerges in early childhood and often persists into adulthood, increasing the risk of poor educational performance, poor employment outcomes, and social, emotional, and behavioral problems (Norbury et al., 2016; Nudel et al., 2023).

Autism spectrum disorder (ASD) is also a highly heterogeneous neurodevelopmental disorder, with a wide range of symptom severity and functional abilities (Georgiou & Spanoudis, 2021). According to the *Diagnostic Manual of Mental Disorders—Fifth Edition—Text Revision* (*DSM-5-TR*), ASD is characterized by persistent difficulties in (1) social interaction and communication in multiple contexts and (2) restricted and repetitive behaviors, interests, and activities (American Psychiatric Association, 2022). ASD symptoms typically emerge in early childhood and persist across multiple contexts throughout an individual's life (Kamp-Becker et al., 2021). The prevalence of ASD has increased in recent years, with current estimates indicating that 1–1.5% of children are affected in Europe (Bougeard et al., 2021).

Children with DLD encounter challenges across various aspects of language acquisition, with formal grammar often being one of the core deficits, especially in early elementary years (Huang & Finestack, 2020). These children typically struggle with a wide range of syntactic structures, particularly those involving noncanonical word order, such as object relative clauses, wh-questions, and passive constructions (Georgiou & Theodorou, 2022). In children with ASD, the core deficits are primarily related to communication, although recent research has identified grammatical deficits as an additional area of concern (Huang & Finestack, 2020). These difficulties may include omissions of functional words, simplified sentence structures, and limited syntactic diversity (Huang & Finestack, 2020). Syntactic difficulties can have a negative impact on several areas of development, including academic achievement, literacy acquisition, mental health, and social integration (Balthazar et al., 2020; Rinaldi et al., 2021)

The extent of linguistic challenges and their negative impact on children's development highlight the importance of early identification of language difficulties in children and subsequent intervention (Rinaldi et al., 2021). Therefore, early, effective, evidence-based interventions are essential to minimize long-term consequences and support children's participation in everyday contexts (Sansavini et al., 2021).

Grammatical intervention for children with syntactic impairment has traditionally relied on implicit approaches, which aim to facilitate grammar acquisition through increased exposure to target structures within meaningful communicative contexts. Techniques such as imitation, focused stimulation, modeling, and conversational recasting are commonly employed (Finestack, 2018; Leonard, 2014; Plante et al., 2014). These approaches are predicated on the assumption that children possess the capacity to discern regularities in linguistic input, independent of explicit instruction (Calder et al., 2017).

Although widely used and often beneficial, many interventions employing implicit-only (I-O) approaches tend to yield moderate effects (Leonard, 2014; Plante et al., 2014). For example, Plante et al. (2014) examined the use of enhanced conversational recasts, primarily an implicit grammar facilitation approach, to improve the production of grammatical targets. This study found a significant treatment effect for the implicit high variability intervention in only six sessions, but the overall gains were modest.

Explicit approaches, on the other hand, are based on metalinguistic principles and provide direct instruction about grammatical rules, typically supported by visual cues, structured tasks, and guided metacognitive reflection (Delage et al., 2024; Ebbels, 2014; Finestack, 2018). These approaches promote awareness of the grammar principles underlying syntactic structures and are particularly recommended for populations with suspected deficits in implicit learning (Klinger et al., 2007).

Emerging literature suggests that interventions that combine implicit and explicit (E-I) approaches are beneficial in helping children with DLD acquire grammatical forms. For example, in a quasi-experimental study, Smith-Lock et al. (2013) examined the effectiveness of an expressive grammar intervention delivered in classroom-based small groups to 34 children with DLD aged 5 years old. The study compared an experimental group (EG) that received a combination of E-I approaches with a control group (CG) that received general language stimulation. The authors reported significant improvements in the EG following 8 weekly 1-hr sessions.

Finestack (2018) conducted a study comparing the acquisition, maintenance, and generalization of three novel grammatical forms (gender marker, tense marker, and the third-person singular marker). Twenty-five children between the ages of 5 and 8 years with DLD were randomly assigned (E-I condition, *n* = 13; I-O condition, *n* = 13) to receive either an I-O or combined E-I intervention. Each participant completed five teaching lessons for each of the three novel grammatical forms. Findings revealed significant learning advantages for the E-I group on each of the three grammatical forms. These findings indicate a clear advantage for explicit techniques when targeting grammatical forms.

The current literature on interventions that specifically target the grammatical skills of children with ASD is scarce, despite evidence of significant deficits in these areas among this developmental condition. The limited investigation that has been conducted seems to indicate that an explicit approach to training grammatical forms may be particularly beneficial for children with ASD as opposed to implicit approaches. Klinger et al. (2007) proposed that deficits in implicit learning for individuals with ASD lead them to use more effortful, explicit approaches to accomplish tasks that appear effortless for typical development children. This suggests a possible link between implicit learning skills and language development in ASD. Additional investigation into intervention approaches in children with ASD was carried out by Bangert et al. (2019), in which an early efficacy study was conducted to examine the effectiveness of an intervention approach that combined implicit modeling and recasting approaches with explicit instruction, in which the pattern guiding the targeted form was directly presented. A total of 17 children with ASD between the ages of 4 and 9 years participated in the study. Results demonstrate that an E-I intervention approach is advantageous when targeting grammatical forms.

In the international literature, some intervention programs tooted in explicit instruction have been developed and described to improve children's syntactic skills, such as Colorful Semantics (Bryan, 1997), Shape Coding (Ebbels, 2007), and MetaTaal (Zwitserlood, 2015). Research on these programs has been conducted to determine their effects on syntactic intervention in children with neurodevelopmental disorders (Calder et al., 2021; Ebbels, 2007, 2014). One such study was conducted by Christopouloua et al. (2021), which examines the effectiveness of Colorful Semantics on the morphosyntactic and semantic development of 40 Cypriot-Greek-speaking children with ASD (EG, *n* = 20; CG, *n* = 20). The intervention lasted 3 months with twice-a-week 45-min sessions in a clinic setting. The main finding of the study is that children who received intervention with Colorful Semantics had better results in mean-length utterances, narratives, and expressive vocabulary.

Another example is the study by Kulkarni et al. (2014), which evaluated the use of Shape Coding combined with recasting and grammar facilitation to improve the use of past tense morphemes with two children with language impairment aged 8; 11 and 9; 4. Both children made statistically significant gains in their use of the target structure in a sentence completion task. A later study conducted by Calder et al. (2018) involved three children aged 6 to 7 years with DLD, who received a combined explicit (Shape Coding) and implicit grammar intervention targeting regular past tense. The intervention was delivered twice a week, with 45-min sessions for 5 weeks, with two of the three participants demonstrating statistically significant improvements in receptive and/or expressive grammar.

Zwitserlood (2015) conducted a within-participant concurrent single-case experimental design study to examine the effects of MetaTaal for improving complex syntax in 13 Dutch-speaking school-age children with DLD. A total of 10 individual therapy sessions lasting 30 min each, twice a week, for 5 weeks were delivered. This study revealed that participants exhibited enhanced proficiency in the production of relative clauses. However, no discernible advancement was noted in the receptive task.

Despite the existing studies, the small number of participants and the type of study design used in the various studies make it difficult to generalize the results obtained (Bangert et al., 2019; Christopouloua et al., 2021), with particular difficulties in generalizing results based on single-subject study designs (Spieth et al., 2016).

In addition to the theoretical orientation, the mode of intervention delivery plays a crucial role in intervention outcomes. Studies show considerable variability in delivery characteristics. For example, the syntactic interventions reviewed by Balthazar et al. (2020) were designed to align with clinical feasibility, particularly within educational or therapeutic settings. The authors report that individual sessions delivered once or twice per week, lasting between 20 and 60 min over periods ranging from 5 to 12 weeks, have demonstrated clinical effectiveness. The intervention approaches were implemented with clearly defined grammatical targets and were characterized by the use of explicit metalinguistic approaches, systematic organization of stimuli, and multimodal representations. The accumulated evidence suggests that this delivery model, even at moderate intensity, can lead to significant linguistic gains, highlighting its practical applicability. This aligns with findings from Calder et al. (2021), who compared two delivery models of the same explicit intervention—once versus twice a week—and found that both schedules led to significant improvements in past-tense production. However, the twice-weekly condition resulted in a greater rate of progression and higher maintenance scores, suggesting the potential benefits of increased cumulative intervention intensity.

Despite these findings, few studies have specifically examined the clinical practices of speech and language therapists (SLTs) regarding syntactic intervention in children with DLD or ASD. One example is the study conducted by Finestack and Satterlund (2018). The authors conducted a large-scale survey with 338 American SLTs and found that, although grammatical intervention is a common component of clinical work, especially targeting early developing structure forms, complex syntactic structures are rarely addressed. Interventions are typically delivered in individual or small-group formats within therapy rooms, with average sessions lasting 29 min and occurring 3 to 4 times per month. The most frequently used procedures included modeling, recasting, imitation, and explicit instruction. More recently, Azevedo et al. (2025) examined the clinical practices of Portuguese SLTs working with preschool-age children with DLD or ASD with a cross-sectional survey with 357 participants. Interventions were predominantly delivered in clinical or school settings, with sessions typically lasting 45–60 min weekly and often involved collaboration with families and early childhood educators (ECEs). Between 89% (*n* = 282 for DLD) and 91% (*n* = 194 for ASD) of the SLTs did not use any structured program or method specifically for syntactic intervention.

In the context of intervention studies targeting development in children with neurodevelopment disorders, standardized language assessments were used to evaluate treatment outcomes, given their psychometric robustness, objectivity, and comparability across participants (Calder et al., 2018; Christopouloua et al., 2021; Delage et al., 2024). In the present study, outcome measures were selected based on their alignment with the intervention targets. Two standardized instruments developed and validated for European Portuguese were employed—SIN:TACS (Vieira, 2018) and Subtest 3 of Avaliação da Linguagem Oral (ALO; Sim Sim, 2006). The SIN:TACS is the only available standardized instrument designed specifically to assess expressive syntactic skills in European–Portuguese-speaking preschool-age children. It comprises structured elicitation tasks that target a wide range of syntactic structures (Vieira, 2018). Subtest 3 of ALO, frequently used in clinical and research settings, evaluates syntactic comprehension (Sim Sim, 2006).

Additionally, although caregivers and teacher questionnaires have been predominantly employed in the assessment of pragmatic abilities, emerging evidence suggests that these tools can also provide valuable insight into other language domains by capturing functional changes in real-life communicative contexts (O'Shea et al., 2023; Pratt et al., 2022).

Considering the assessment process, Finestack and Satterlund (2018) found that American SLTs most frequently used observational methods, informal language samples, and informal probes to monitor grammatical progress. Although standardized assessments were also used, naturalistic measures were generally preferred. Azevedo et al. (2025) reported that most Portuguese SLTs used a combination of formal and informal assessment tools when working with children with ASD (74%) and DLD (79%). However, fewer than 10% reported using tools specifically designed to assess syntax, such as SIN:TACS. Additionally, a considerable proportion of SLTs reported low or very low confidence in their ability to assess and intervene with preschool-age children with syntactic impairment, which could reinforce the need for accessible, structured, and validated programs to guide clinical practice (Azevedo et al., 2025).

In Portugal, two syntactic intervention programs have been developed and content-validated for European Portuguese: *INsyntax* (Lopes et al., 2022, 2023) and *PROsyntax* (Azevedo et al., 2021). PROsyntax is an intervention program based on the principles of explicit metalinguistic approaches, including tasks that aim to explain to the child the grammatical rules underlying each syntactic structure using a specific visual coding system (Azevedo et al., 2021). This system consists of a set of colors and symbols to represent syntactic categories and rules, thus facilitating the comprehension and manipulation of grammatical structures. The program encompasses a wide range of syntactic structures mentioned in the literature as being problematic for children with language disorders (Durrleman & Delage, 2016; Santos & Martins, 2022). It includes (1) sentence structure, (2) agreement, (3) pronouns, (4) coordinated clauses, (5) subordinated clauses (adverbial and complement), (6) passives, and (7) wh-questions and relative clauses (Azevedo et al., 2021). Two preliminary studies have examined the two syntactic programs. Marques et al. (2023) conducted a pilot pre–post study to investigate the effects of PROsyntax in a sample of seven children with DLD, while Martins et al., 2023 carried out a similar pilot pre–post study involving five children with ASD who received intervention with the INsyntax program. Although promising outcomes were reported, methodological limitations constrain the strength and generalizability of the findings. These include small sample sizes, the absence of a control group, and short intervention duration. These limitations highlight the need for further controlled studies with greater methodological robustness.

Accordingly, the present study aims to determine the effects of PROsyntax in preschool-age children diagnosed with DLD or ASD, all of whom present syntactic impairments. The following research questions are raised: (1) Does the PROsyntax lead to significant improvements in expressive and receptive syntactic skills in preschool-age children with DLD?; (2) Does the PROsyntax lead to significant improvements in expressive and receptive syntactic skills in preschool-age children with ASD?; (3) Are gains in expressive syntactic skills significantly associated with gains in receptive syntactic skills in these populations?

## Methods

The following report of this study is guided by the Transparent Reporting of Evaluations with Non-randomized Designs (TREND) Statement (Des Jarlais et al., 2004). The TREND Statement checklist can be consulted in the Supplemental Material.

### Design

A nonrandomized controlled trial with a nonprobabilistic sample was conducted to determine the effects of the *PROsyntax* in preschool-age children diagnosed with DLD and children diagnosed with ASD with syntactic impairment. This study was approved by the Ethics Committee for Research of the Faculty of Letters of the University of Lisbon (number 10_CEI2022).

Several educational institutions in the district of Aveiro, Portugal, were contacted to participate in the project. Written authorization from the schools was obtained after a thorough presentation of the aims of the study and a detailed description of the intervention procedures. Written informed consent was obtained from the parents before data collection and after receiving a detailed explanation of the study.

The community members were not involved in the study.

### Participants

Upon obtaining authorization from the participating early childhood institutions in the district of Aveiro, Portugal, the recruitment process was initiated. ECEs were provided with detailed information regarding the eligibility criteria for identifying children with the potential to be included in the study.

The children were recruited based on predefined inclusion and exclusion criteria. The inclusion criteria were: (1) diagnosis of DLD (children were diagnosed after an assessment with Teste de Linguagem—Avaliação da Linguagem Pré-Escolar (TL-ALPE; Mendes, Lousada et al., 2014) applied by an SLT blind to the aims of the study) or ASD (clinical diagnosis provided by the neurodevelopmental pediatrician according to *DSM-V* criteria, ADOS, and/or ADI-R); (2) the presence of syntactic impairment (confirmed through a language assessment with TL-ALPE (Mendes et al., 2014) applied by an SLT blind to the aims of the study); (3) aged between 3;0 and 6;11 years and; and (4) European Portuguese as a native language. The exclusion criteria were (1) the presence of an intellectual developmental disorder and (2) bilingualism.

**Figure 1.**
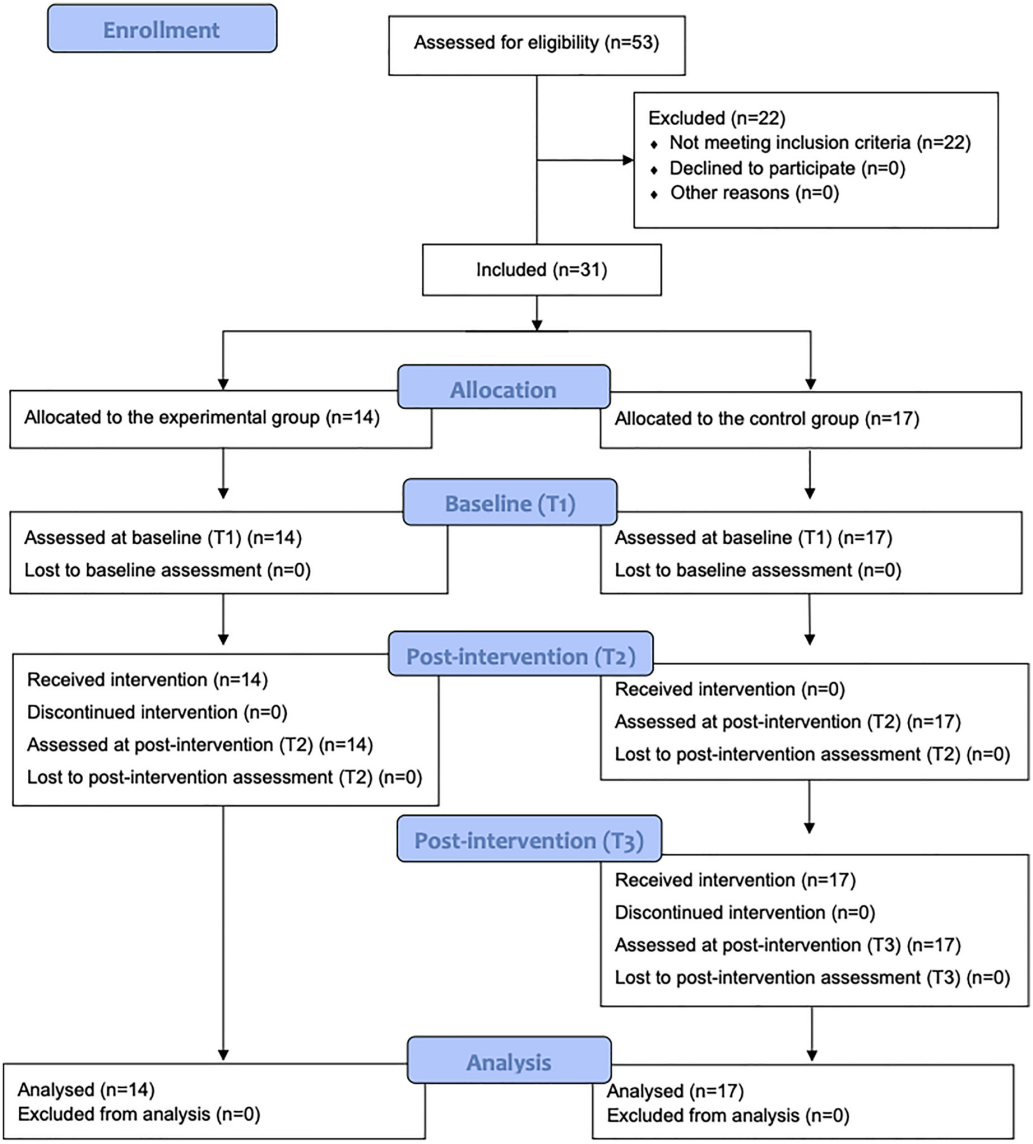
CONSORT flow diagram.

Figure 1 shows the enrollment diagram based on CONSORT (Schulz et al., 2010). A priori power analysis was performed using G*Power version 3.1.9.6 (Faul et al., 2007) to determine the required sample size for comparing two groups of independent samples. Assuming a large effect size (1.29), an α = .05 (two-tailed), and a power of .95, the required sample size was estimated to be 31 participants. As such, a total of 31 eligible children were identified and subsequently assigned to either the EG (intervention) (*n* = 14) or the CG (without intervention) (*n* = 17).

Although a randomized design was initially planned, it proved unfeasible due to ethical and logistical constraints in the implementation context. Specifically, participants were identified progressively by early childhood educators across several early childhood institutions, and it was not possible to allocate all children at the same time. For this reason, a nonrandomized design was adopted. This methodological approach has been employed in other recent studies involving these populations (e.g., Pereira et al., 2025).

To enhance the internal validity of the nonrandomized design, baseline equivalence between groups was statistically assessed for age, sex, and preintervention syntactic performance through statistical tests before outcome analysis.

**Figure 2.**
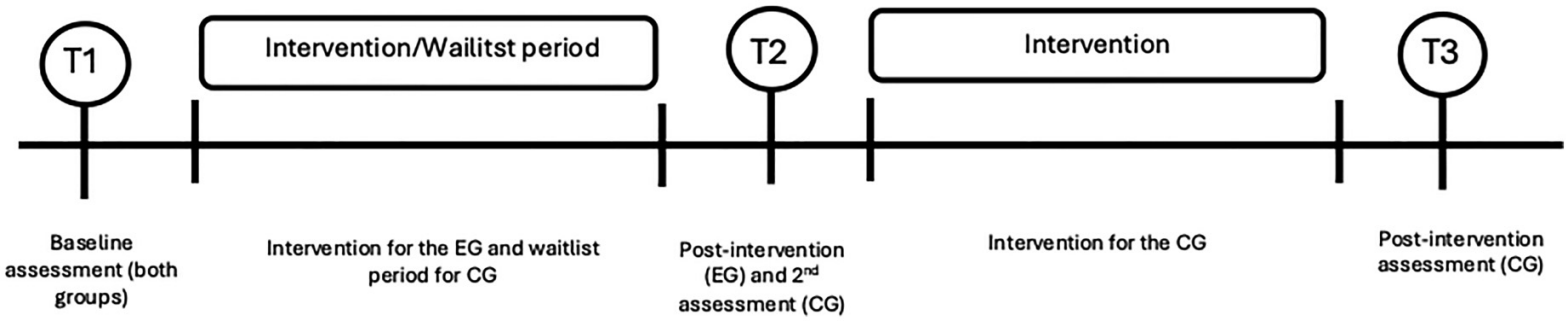
Overview of the assessment and intervention phases for experimental and control groups.

Figure 2 provides an overview of the assessment and intervention phases for each group. All participants completed a baseline assessment (T1) immediately after the group allocation. The EG underwent the intervention period, during which the CG remained on a waitlist. A second assessment (T2—postintervention for EG) was conducted for both groups 1 week after the EG's intervention ended. Subsequently, the CG received the same intervention, followed by a final assessment (T3—postintervention for CG). Both assessments and the intervention were carried out at the children's educational institution.

### Intervention

The Template for Intervention Description and Replication (Hoffmann et al., 2014) was used to ensure a clear and replicable description of the intervention in this study. The checklist can be consulted in the Supplemental Material.

The intervention in this study was based on PROsyntax, which aimed to improve syntactic skills in preschool-age children with syntactic impairments. The program's content was validated for European Portuguese through a content validation process, followed by a pilot study to assess its acceptability. This is a manualized intervention program that follows the principles of a metalinguistic approach while incorporating elements from internationally recognized programs, such as Shape Coding and Colorful Semantics. The selection of syntactic structures included in the program was based on both structures from established international programs and those identified in the literature as crucial for syntactic acquisition. The program employs, as is common in other international programs, a visual coding system consisting of images and symbols to present linguistic processes, such as agreement aspects and grammatical rules. The manual includes detailed objectives, activities, procedures, strategies, and supplementary materials that are useful for the implementation of the intervention. For each objective, the activities first involve the explicit presentation of linguistic knowledge, where the child is taught the grammatical rules underlying each structure. Following this phase, there are tasks aimed at exposing the child to stimuli that support the training of both comprehension and production of the targeted syntactic structures.

The PROsyntax provides physical materials, such as illustrations representing the target sentence. Additionally, it is recommended to use other materials (e.g., objects chosen according to the preferences of each child) to further support the intervention on the target syntactic structures. Furthermore, the program includes a checklist of syntactic structures, which the SLT is required to complete based on the child's ability to produce or comprehend each structure included in the program. A document is also provided, which can be distributed to ECEs and/or families, offering strategies and activity suggestions for implementation in their respective contexts.

Despite the use of PROsyntax, the intervention was tailored to each child. Intervention objectives were collaboratively defined with ECEs and families, using the checklist provided in the program to prioritize and set goals according to the specific needs of each child. No modifications were made to the intervention during its course.

The intervention was implemented by an SLT (first author) with in-depth knowledge of the program's content and its clinical implementation, along with prior clinical experience in working with children with syntactic impairments in educational settings. The intervention was delivered in a naturalistic (early childhood institution) context, in a dedicated space for this purpose. It was conducted on an individual, face-to-face basis, with all children receiving the same number of sessions (24), each lasting 1 hr. Sessions were provided twice a week, free of charge. Throughout the implementation process, continuous contact with ECEs and families was maintained to promote the use of strategies and activities, ensuring the generalization of the target syntactic structures. This regular contact was also maintained to promote adherence to the implementation of the project.

All children in the CG remained on a waiting list until the T2 assessment (postintervention of the EG). After that, the same intervention previously provided to the EG was applied to the CG. All children identified and assigned to the groups completed the intervention as planned. The data was collected between September 2023 and June 2024.

### Outcome Measures

#### Primary outcome measure

The primary outcome measure used was SIN:TACS—Teste de Avaliação da Competência Sintática (Vieira, 2018). The SIN:TACS is a standardized assessment tool valid and reliable for European Portuguese, and it consists of 40 items, each scored dichotomously (0 for *incorrect* and 1 for *correct*), for a maximum total score of 40 points. It aims to assess children aged 3 to 6 years regarding the production of several syntactic structures, mainly through elicitation techniques (Vieira, 2018).

This instrument presents an internal consistency (Lambda-2 coefficient) of 0.87. Test–retest reliability, assessed with a 2-month interval, was satisfactory (*r*_a_ = .80; *p* < .01). Construct validity was confirmed through significant correlations with another standardized language test (*r* = .67; *p* < .01), and score validity was further supported by significant age differentiation (*r* = .66; *p* < .01). This assessment test was considered a valid and reliable tool to assess syntactic development in clinical and research contexts (Vieira, 2018). Raw scores were used for all analyses.

#### Secondary outcome measure

The second outcome measure was Subtest 3 of ALO (Sim Sim, 2006). This subtest is part of a standardized assessment tool for European Portuguese designed to assess various linguistic domains in preschool and school-age children. Subtest 3 is designed to assess comprehension of complex syntactic structures and involves the child's ability to answer a question based on a proposed statement—a statement that contains different syntactic structures to be assessed (Sim Sim, 2006).

The selected subtest comprises 32 items, each scored dichotomously, with a maximum total score of 32 points. Internal consistency (Cronbach's alpha) yielded a value of .90. Raw scores were used for all analyses.

#### Assessment procedures

All assessments were conducted by an SLT, blind to the aims of the study as well as to the participants’ group allocation, with prior experience in the assessment of children with syntactic difficulties and familiarity with the administration of the instruments used in this study. Assessments took place individually in an appropriate room within the child's early childhood institution. These measures were applied consistently across all assessments (T1, T2, and T3), and the same procedures were followed at each time point.

#### Satisfaction questionnaires

A satisfaction questionnaire was developed to gather information about the perceived feasibility and acceptability of the intervention from the perspective of the children's families and ECEs. The questions addressed the following: (1) perceived changes in the child's expressive language; (2) perceived changes in the child's receptive language; (3) interaction and participation in the familiar and educational contexts; and (4) overall satisfaction with the project. This questionnaire was used only during the T2 assessment with the EG. Responses ranged from 0 to 4, where 0 indicated “*no improvement/very dissatisfied*” and 4 indicated “*significant improvement/extremely satisfied*.” Families and ECEs completed the questionnaire independently with the opportunity to clarify any doubts if needed.

#### Data analysis

Statistical analysis was conducted using the Statistical Package for Social Sciences software (IBM SPSS Statistics v29.0) and comprised descriptive and inferential statistics. The unit of analysis was the individual child.

Descriptive statistics included the calculation of means (M) and standard deviation (SD) for quantitative variables, and percentages (%) for qualitative variables. These were used to characterize the sample in terms of sociodemographic and clinical variables, as well as to describe baseline performance. For ordinal variables from the questionnaire, the median (Mdn) and interquartile range (IQR; 25th and 75th percentiles) were calculated.

Inferential statistics began with the assessment of group comparability at baseline. Independent-sample *t*-tests were used for continuous variables, and chi-square or Fisher's tests were applied to categorical variables. The normality of distributions was verified using the Shapiro-Wilk test.

To evaluate the effect of the intervention, repeated-measures ANOVAs (three-way mixed ANOVAs) were conducted, with the factors (Time: baseline and postintervention, Group: experimental and control, and Condition: DLD and ASD). Mauchly's test was used to assess the assumption of sphericity, and Levene's test was used to verify the homogeneity of variances. Effect sizes were calculated using partial eta squared (η_p_²), interpreted according to the guidelines established by Cohen (1988) and Sawilowsky (2009). The effect size of Cohen's (d) and post hoc power calculations was determined using G*Power version 3.1.9.6 (Faul et al., 2007).

To examine whether improvements in syntactic production were associated with improvements in syntactic comprehension, a correlation analysis was conducted between gains (T2-T1) in SIN:TACS and ALO scores using Spearman's rank correlation coefficient. Correlations between outcome measures as well as demographic variables were also employed.

Moreover, differences between families and ECEs’ ratings were analyzed using Wilcoxon signed-rank tests. The level of significance used was 0.05.

## Results

### Sociodemographics and Baseline Characteristics

Table 1 presents the sociodemographic and clinical characteristics of the participants at baseline (T1). The participants were from the same demographic region, attended early childhood institutions, and shared a similar socioeconomic background. There were no statistically significant differences between groups in both categorical and continuous variables. Additionally, considering the age range of the participants in both groups, the expected normative means and standard deviations for the outcome measures were 22.78 ± 5.11 for SIN:TACS (Vieira, 2018) and 14.7 ± 4.5 for Subtest 3 of ALO (Sim Sim, 2006).

**Table 1.** Characteristics of the Participants at Baseline.

Categorial variables	Experimental group (*n* = 14)		Control group (*n* = 17)		Statistical results
	*n*	(%)	*N*	(%)	
Condition					
DLD	9	(64.28)	10	(58.82)	χ2 (1) = 0.09; *p* = .756
ASD	5	(35.71)	7	(41.17)	
Sex					
Male	10	(71.43)	12	(70.59)	Fisher = 0.00; p = 0.637
Female	4	(28.57)	5	(29.41)	
Continuous variables	M ± SD				
Age (months)	58.50 ± 9.91		58.82 ± 8.96		t(29)=-0.09; p = 0.925
SIN:TACS	12.14 ± 6.88		10.88 ± 5.32		t(29) = 0.58; p = 0.285
Subtest 3 of ALO	7.86 ± 4.57		7.12 ± 5.15		t(29) = 0.41; p = 0.339

ASD: autism spectrum disorder; DLD: developmental language disorder; M: mean; SD: standard deviation; SIN:TACS: Teste de Avaliação da Competência Sintática (Vieira, 2018); Subset 3 of ALO: Subtest 3 of Avaliação da Linguagem Oral (Sim Sim, 2006).

### Outcome Measures

The data about the primary and secondary outcome measures before and after the intervention of the EG can be found in Table 2*.*

**Table 2.** Results of SIN:TACS and Subtest 3 of ALO (Three-Mixed Factors ANOVA)—Postintervention of the EG.

	Experimental group (*n* = 14)			Control group (*n* = 17)			Statistical results
	Baseline (T1)	Postintervention (T2)	Difference (T2 − T1)	Baseline (T1)	Postintervention (T2)	Difference (T2-T1)	
	M ± SD	M ± SD	M ± SD	M ± SD	M ± SD	M ± SD	
SIN:TACS							
Condition							
DLD (*n* = 19) ASD (*n* = 12)	15.89 ± 5.48 5.40 ± 2.30	27.67 ± 6.21 19.40 ± 2.78	11.78 ± 2.86 14.00 ± 1.22	12.40 ± 4.99 8.71 ± 5.38	14.00 ± 5.62 10.57 ± 4.96	1.60 ± 0.97 1.86 ± 0.69	F_T_(1,27) = 503.02; p < 0.001; η_p_^2^ = 0.95 F_G_(1,27) = 9.01; p < 0.001; η_p_^2^ = 0.89 F_C_(1,27) = 11.74; p = 0.006; η_p_^2^ = 0.25 F_T×G_(1,27) = 293.22; p < 0.001; η_p_^2^ = 0.92 F_T×C_(1,27) = 3.62; p = 0.068; η_p_^2^ = 0.12 F_G×C_(1,27) = 2.38; p = 0.135; η_p_^2^ = 0.08 F_T×G×C_(1,27) = 2.27; p = 0.014; η_p_^2^ = 0.08
ALO							
Condition							
DLD (*n* = 19) ASD (*n* = 12)	10.33 ± 3.54 3.40 ± 2.07	21.56 ± 3.97 14.60 ± 1.14	11.22 ± 3.42 11.20 ± 2.59	7.80 ± 4.05 6.14 ± 6.64	9.20 ± 3.99 7.14 ± 7.05	1.40 ± 0.84 1.00 ± 1.00	F_T_(1,27) = 226.21; p < 0.001; η_p_^2^ = 0.89 F_G_(1,27) = 9.03; p = 0.006; η_p_^2^ = 0.25 F_C_(1,27) = 7.28; p = 0.012; η_p_^2^ = 0.12 F_T×G_(1,27) = 147.18; p < 0.001; η_p_^2^ = 0.85 F_T×C_(1,27) = 0.07; p = 0.800; η_p_^2^ = 0.00 F_G×C_(1,27) = 2.43; p = 0.130; η_p_^2^ = 0.08 F_T×G×C_(1,27) = 0.05; p = 0.821; η_p_^2^ = 0.00

T1: baseline assessment; T2: postintervention assessment; T: time; G: group; C: condition; M: mean; SD: standard deviation; SIN:TACS: Teste de Avaliação da Competência Sintática (Vieira, 2018); ALO: Avaliação da Linguagem Oral (Sim Sim, 2006).

The mean and standard deviation were calculated for the results obtained at T1 and T2 for both groups, as well as the mean and standard deviation for the difference between the two time points (T2 − T1).

The primary outcome measure was SIN:TACS (Vieira, 2018). A three-mixed factors ANOVA revealed a significant and large effect of Time × Group interaction (F_T × G_(1,27) = 293.22; *p* < .001; η_p_^2^ = 0.92). The Time × Condition and Group × Condition interactions were not significant (F_T × C_(1,27) = 3.62; *p* = .068; η_p_^2^ = 0.12; F_G × C_(1,27) = 2.38; *p* = .135; η_p_^2^ = 0.08, respectively). However, the Time × Group × Condition interaction reached significance (F_T × G × C_(1,27) = 2.28; *p* = .014; η_p_^2^ = 0.08) with a medium effect size.

The secondary outcome measure was Subtest 3 of ALO (Sim Sim, 2006). The interaction between time and group was statistically significant with a large effect size (F_T × G_(1,27) = 147.18; *p* < .001; η_p_^2^ = 0.85). The Time × Condition interaction was not significant (F_T × C_(1,27) = 0.06; *p* = .800; η_p_^2^ = 0.002), nor was the Group × Condition interaction (F_G × C_(1,27) = 2.43; *p* = .130; η_p_^2^ = 0.08). The three-way interaction (Time × Group × Condition) was also not significant (F_T × G × C_(1,27) = 0.52; *p* = .821; η_p_^2^ = 0.002) with a negligible effect size.

The results showed huge effect sizes (d > 2.00) for both outcome measures, based on the differences (T2-T1) (SIN:TACS, d = 4.07 (DLD) and d = 11.67 (ASD); ALO, d = 3.29 (DLD) and d = 4.31 (ASD)). Post hoc power analysis indicated a power of 1.00 for all measures and conditions.

Correlation analysis related to sociodemographic variables and outcome measures is presented in Table 3. A strong positive correlation was found between performance on the primary and secondary outcome measures at both baseline (T1: *r* = .86; *p* < .001 (DLD); *r* = .82; *p* < .001 (ASD); *r* = .81; *p* < .001 (Total)) and postintervention (T2: *r* = .82; *p* < .001 (DLD); *r* = .93; *p* < .001 (ASD); *r* = .88; *p* < .001 (Total)). No significant correlation was observed between T1 and differences (T2 − T1) for either SIN:TACS (*r* = .16; *p* = .503 (DLD); *r* = .25; *p* = .443 (ASD); *r* = −.003; *p* = .986 (Total) or Subtest 3 of ALO (*r* = −.004; *p* = .881 (DLD); *r* = −.22; *p* = .487 (ASD); *r* = −.11; *p* = .572 (Total)). Concerning sociodemographic characteristics, no significant association was found between age or sex and outcome measures, either overall or within the condition.

**Table 3.** Correlation Analyses Between Outcome Measures and Demographic Variables.

Correlations	DLD (*n* = 19)	ASD (*n* = 12)	Total (*n* = 31)
Outcome measures			
SIN:TACS (preintervention) x ALO (preintervention)	*r* = .86; *p* < .001	*r* = .82; *p* < .001	*r* = .81; *p* < .001
SIN:TACS (postintervention) x ALO (postintervention)	*r* = .82; *p* < .001	*r* = .93; *p* < .001	*r* = .88; *p* < .001
SIN:TACS (T1) x Difference SIN:TACS	*r* = .16; *p* = .503	*r* = .25; *p* = .443	*r* = −.00; *p* = .986
ALO (T1) x Difference ALO	*r* = −.04; *p* = .881	*r* = −.22; *p* = .487	*r* = −.11; *p* = .572
Demographic variables			
Age x Difference ALO	*r* = .18; *p* = .466	*r* = −.09; *p* = .789	*r* = .08; *p* = .697
Age x Difference SIN:TACS	*r* = .40; *p* = .094	*r* = .26; *p* = .416	*r* = .26; *p* = .160
Sex x Difference SIN:TACS	*r* = .21; *p* = .389	*r* = .23; *p* = .475	*r* = .02; *p* = .927
Sex x Difference ALO	*r* = −.12; *p* = .617	*r* = −0.18; *p* = .585	*r* = −.14; *p* = .451

Preintervention: T1 for EG and T2 for CG; postintervention: T2 for EG and T3 for CG; difference: T2 − T1 for EG and T3 − T2 for CG; DLD: developmental language disorder; ASD: autism spectrum disorder; r: Spearman's rank correlation coefficient; SIN:TACS: Teste de Avaliação da Competência Sintática (Vieira, 2018); ALO: Subtest 3 of Avaliação da Linguagem Oral (Sim Sim, 2006).

Following the postintervention assessment (T2), the CG subsequently received the same intervention. To compare the effects of the intervention across both groups, pre- and postintervention data were analyzed using the same outcome measures. Given that all participants had undergone the same intervention, no statistically significant differences were expected between groups.

Table 4 shows the results of SIN:TACS and Subtest 3 of ALO before and after the intervention in both groups. The differences (T3 − T2 for CG and T2 − T1 for EG) are also provided. Interaction effects were examined through a three-way mixed ANOVA, considering time, group, and condition as factors. For both outcome measures, no significance was observed for the Time × Group × Condition interaction (F_T × G × C_(1,27) = 0.30; *p* = .582; η_p_^2^ = 0.01 (SIN:TACS); F_T × G × C_(1,27) = 0.52; *p* = .821; η_p_^2^ = 0.002 (ALO).

**Table 4.** Results of SIN:TACS and Subtest 3 of ALO before and after Intervention in Both Groups.

	Experimental group (*n* = 14)			Control group (*n* = 17)			Statistical results
	Baseline (T1)	Postintervention (T2)	Difference (T2 − T1)	Before intervention (T2)	After intervention (T3)	Difference (T3-T2)	
	M ± SD	M ± SD	M ± SD	M ± SD	M ± SD	M ± SD	
SIN:TACS							
Condition							
DLD (*n* = 19) ASD (*n* = 12)	15.89 ± 5.48 5.40 ± 2.30	27.67 ± 6.21 19.40 ± 2.78	11.78 ± 2.86 14.00 ± 1.22	14.00 ± 5.62 10.57 ± 4.96	24.50 ± 6.42 22.29 ± 7.48	10.50 ± 1.90 11.71 ± 3.04	F_T_(1,27) = 703.88; *p* < .001; η_p_^2^ = 0.96 F_G_(1,27) = 0.13; *p* < .719; η_p_^2^ = 0.01 F_C_(1,27) = 8.74; *p* = .006; η_p_^2^ = 0.25 F_T×G_(1,27) = 3.88; *p* = .127; η_p_^2^ = 0.13 F_T×C_(1,27) = 3.61; *p* = .068; η_p_^2^ = 0.12 F_G×C_(1,27) = 2.5; *p* = .124; η_p_^2^ = 0.086 F_T×G×C_(1,27) = 0.31; *p* = .582; η_p_^2^ = 0.01
ALO							
Condition							
DLD (*n* = 19) ASD (*n* = 12)	10.33 ± 3.54 3.40 ± 2.07	21.56 ± 3.97 14.60 ± 1.14	11.22 ± 3.42 11.20 ± 2.59	9.20 ± 3.99 7.14 ± 7.06	19.00 ± 4.83 17.0 ± 7.48	9.80 ± 1.75 9.86 ± 2.61	F_T_(1,27) = 457.07; *p* < .001; η_p_^2^ = 0.94 F_G_(1,27) = 0.13; *p* = .723; η_p_^2^ = 0.00 F_C_(1,27) = 6.86; *p* = .004; η_p_^2^ = 0.20 F_T×G_(1,27)= 1.97; *p* = .171; η_p_^2^ = 0.07 F_T×C_(1,27) = 0.00; *p* = .968; η_p_^2^ = 0.00 F_G×C_(1,27) = 2.1; *p* = .163; η_p_^2^ = 0.07 F_T×G×C_(1,27) = 0.52; *p* = .821; η_p_^2^ = 0.00

T1: baseline assessment; T2: postintervention assessment for EG; T3: postintervention assessment for CG; T: time; G: group; C: condition; M: mean; SD: standard deviation; SIN:TACS: Teste de Avaliação da Competência Sintática (Vieira, 2018); ALO: Avaliação da Linguagem Oral (Sim Sim, 2006).

### Satisfaction Questionnaires

**Graph 1.**
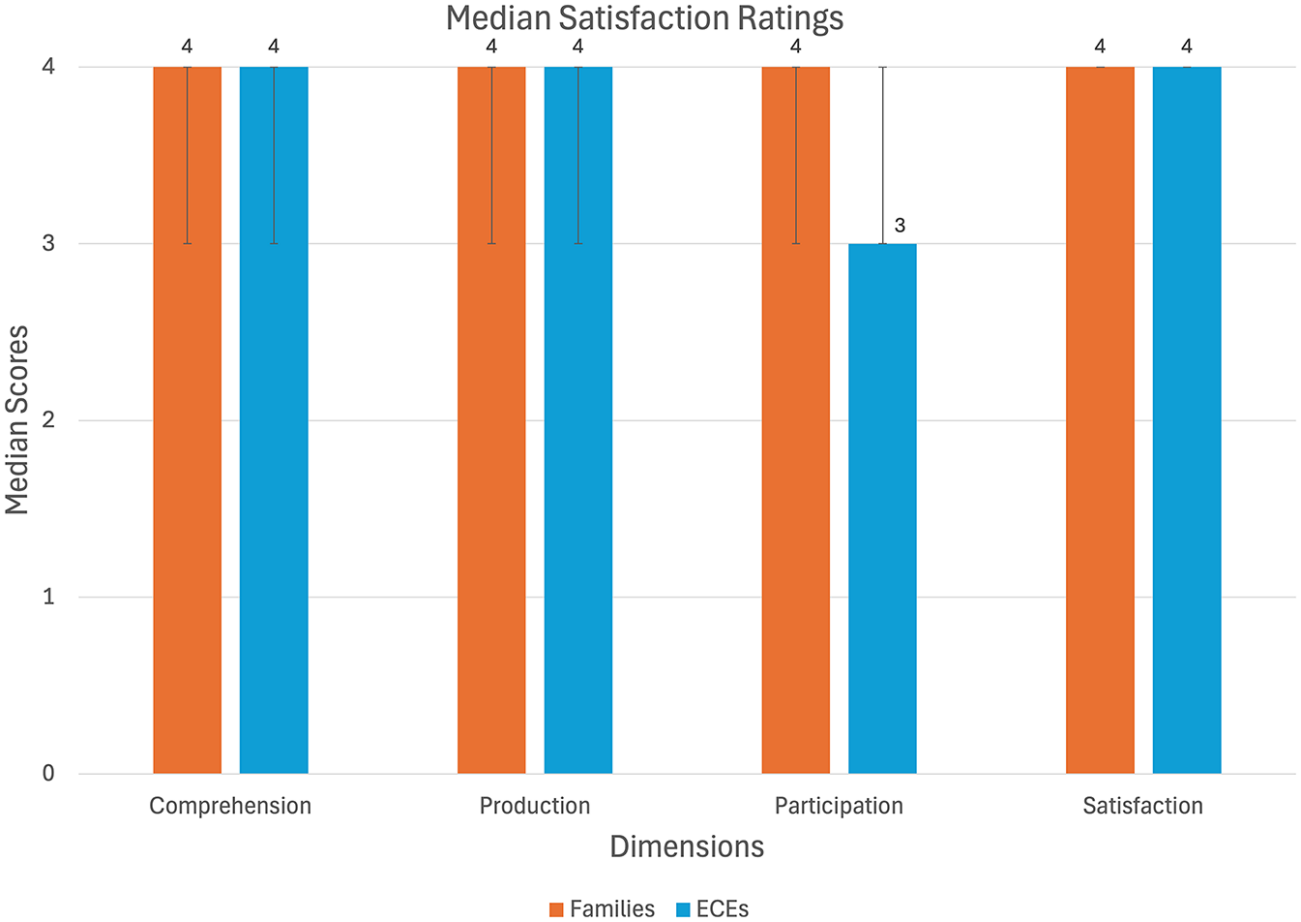
Median satisfaction ratings provided by families and ECEs across four dimensions: comprehension, production, participation in daily contexts, and overall satisfaction. Error bars represent the interquartile range.

Graph 1 shows the median satisfaction ratings for each dimension from both families and ECEs, and IQR ranges are represented by error bars. Satisfaction data were collected from the families and ECEs of the children in the EG (*n* = 14), using a four-point ordinal scale to rate four dimensions: comprehension, production, participation in family/educational contexts, and overall satisfaction with the intervention. Descriptive analysis showed a consistently high level of satisfaction in both groups of informants.

For families, the median range was 4.00 for all dimensions (comprehension: P25 = 3.00; P75 = 4.00; production: P25 = 3.00; P75 = 4.00; participation: P25 = 3.00; P75 = 4.00; satisfaction: P25 = 4.00; P75 = 4.00). For ECEs, the medians were 4.00 for comprehension (P25 = 3.00; P75 = 4.00), production (P25 = 3.00; P75 = 4.00), and satisfaction (P25 = 4.00; P75 = 4.00), and 3.00 for participation (P25 = 3.00; P75 = 4.00). To compare the two groups of informants, a Wilcoxon signed-rank test was conducted for each dimension. No statistically significant differences were found between families and ECEs (comprehension: *Z* = −0.45; *p* = .655; production: *Z* = 0.00; *p* = 1.00; participation: *Z* = −0.15; *p* = .317; satisfaction: *Z* = −0.58; *p* = .564).

## Discussion

This study aimed to determine the effects of the *PROsyntax* program in preschool-age children diagnosed with DLD and children diagnosed with ASD, all presenting syntactic impairment. Using a nonrandomized controlled trial design, the effects of the intervention were assessed through standardized outcome measures of expressive and receptive syntax. The results demonstrated statistically significant improvements in both outcome measures following the intervention. These gains were complemented by consistently high satisfaction ratings from families and ECEs.

A total of 31 children were recruited from early childhood institutions in the Aveiro district (Portugal) and allocated to either the EG or CG based on practical and ethical considerations. All participants met strict inclusion criteria, including a confirmed diagnosis of DLD or ASD and the presence of syntactic impairment. Baseline equivalence between groups was established for age, sex, and preintervention syntactic performance. The sample comprised children aged between 3 and 6 years, and no significant group differences were found in categorical or continuous variables before the intervention, supporting the internal validity of the comparisons drawn.

The primary outcome measure was the SIN:TACS (Vieira, 2018), a standardized tool designed to assess expressive syntactic abilities in European Portuguese-speaking children aged 3 to 6 years. A significant interaction between time and group was observed, indicating that children in the EG exhibited substantial gains in expressive syntax following the PROsyntax intervention. When analyzed by diagnostic condition, children with ASD demonstrated particularly large improvements (*d* = 11.67), while children with DLD showed large gains (*d* = 4.07), suggesting robust effects of the intervention across both groups. Although children with DLD achieved significantly higher absolute postintervention scores, the proportionally greater gains observed in children with ASD, combined with lower baseline scores and reduced variability in postintervention performance, resulted in a larger effect size. These findings are in line with the literature supporting explicit and metalinguistic approaches for children with syntactic impairments in both populations (Bangert et al., 2019; Finestack, 2018). Finestack (2018) found that children with DLD aged 5 to 8 years showed significantly greater gains in novel grammatical forms when taught with an E-I approach. Similarly, Bangert et al. (2019) observed that children with ASD aged 4 to 9 benefited from explicit teaching when this was combined with implicit strategies. Both studies reported effect sizes ranging from medium to large, though lower than those observed in the present study, which may be attributable to differences in intervention dosage and format (Balthazar et al., 2020).

Previous intervention studies that applied programs such as Shape Coding (Calder et al., 2020; Ebbels, 2014) and Colorful Semantics (Christopouloua et al., 2021) reported moderate-to-large improvements in expressive morphosyntax. However, many of these studies targeted specific structures or involved older children, which limits their generalizability to the preschool-age population. In contrast, PROsyntax covers a broad range of syntactic targets and was designed specifically for early childhood implementation, which may also have contributed to the scale of observed effects.

Furthermore, the strong effects found in the ASD group are particularly encouraging, as syntactic intervention in this population remains underresearched. The present findings suggest that explicitly taught syntactic structures can be successfully acquired by children with ASD, especially when visual supports and structured guidance are provided (Bangert et al., 2019; Christopouloua et al., 2021).

The secondary measure was Subtest 3 of ALO (Sim Sim, 2006), a standardized tool designed to assess comprehension of complex syntactic structures in preschool-age children. Similar to the expressive outcomes, a significant interaction between time and group was found, indicating substantial gains in receptive grammar in the EG following the intervention. The analysis by condition (ASD vs. DLD) revealed a large effect size in both populations (*d* = 4.31 for ASD; *d* = 3.29 for DLD), suggesting that children in both groups benefited from the intervention.

These results contribute to a growing, though still limited, evidence of the effects of explicit instruction on receptive grammar. A few studies have shown promising effects on comprehension. Calder et al. (2018) reported moderate improvements in receptive grammar following an intervention that combined Shape Coding with a systematic cueing hierarchy in a quasi-experimental pre–post study involving six children with DLD. In a subsequent single-case experimental study, only one of nine participants showed a sustained improvement in receptive grammar (Calder et al., 2020). Similarly, a randomized controlled trial revealed no significant group-level effects on receptive outcomes, suggesting that the initial positive findings may not reflect consistent treatment efficacy (Calder et al., 2021).

These findings align with the broader literature, which highlights the challenges of effecting measurable change in receptive syntax through intervention. For example, Ebbels (2014) noted limited evidence for intervention effects on receptive grammar, and Finestack (2018) suggested that receptive language may function more reliably as a predictor of grammatical intervention success than as an outcome in itself. Taken together, these studies indicate that although receptive grammar can be targeted in intervention, consistent and generalizable gains remain difficult to achieve. Given this context, the current findings are notable. The use of structured visual supports, explicit rule teaching, and child-centered pacing in PROsyntax may have facilitated receptive gains by reinforcing comprehension through multiple modalities. These elements are likely to be especially beneficial for children with ASD, who often rely more heavily on visual processing in learning contexts (Bangert et al., 2019).

Correlation analyses were conducted to explore the potential influence of participant characteristics and baseline performance on intervention outcomes. The correlational analysis between the primary and secondary outcome measures revealed strong positive associations across conditions at both baseline and postintervention. Despite Finestack's (2018) view, the current findings support that both domains can benefit concurrently from a structured and explicit approach. This relationship reinforces the importance of assessing and addressing both receptive and expressive components of syntax within interventions, particularly in populations such as ASD and DLD, where language profiles are highly heterogeneous.

However, baseline performance on either outcome measure did not significantly predict gains following intervention, suggesting that the progress was not determined by initial skill level. Furthermore, no significant associations were observed between age or sex and the intervention outcomes. These findings support the interpretation that the observed gains were primarily attributable to the intervention itself rather than being confounded by sociodemographic variables or baseline language ability.

PROsyntax intervention was delivered individually, twice per week, over a total of 24 sessions—a relatively intensive dosage compared to other studies (Christopouloua et al., 2021; Zwitserlood, 2015). Evidence supports the association between higher-intensity interventions and stronger treatment effects in grammatical development (Balthazar et al., 2020). While Frizelle et al. (2021) note that higher dosages may lead to diminishing returns beyond a certain threshold, the present study aligns with findings suggesting that structured, intensive, and visually supported explicit instruction can be beneficial for syntactic acquisition—particularly when the intervention is tailored to individual needs and delivered over a well-defined period.

Following the intervention with the EG to assess the replicability of these findings, the same intervention was subsequently delivered to the CG. Comparative analysis of pre- and postintervention scores (T3 − T2) in the CG mirrored the results previously observed in the EG, with significant improvements observed in both outcome measures. Specifically, gains in SIN:TACS and ALO were substantial and consistent with those found in the EG, further supporting the replicability of the intervention effects. Three-way mixed ANOVAs revealed no significant interactions between group, condition, and time, confirming the comparability of the intervention's impact across both implementations.

The similar patterns of improvement observed in both groups, despite their sequential implementation, provide further evidence of the effectiveness and robustness of PROsyntax. These results also highlight the feasibility of implementing the program with fidelity across different cohorts. The use of the same SLT in both phases may have contributed to treatment consistency while also minimizing variability related to implementation.

In addition to standardized outcome measures, a satisfaction questionnaire was administered to families and ECEs to evaluate perceived changes in children's syntactic skills and overall satisfaction with the intervention. This measure provided valuable insight into functional gains observed in naturalistic contexts and complemented the quantitative assessment data. Results indicate high levels of satisfaction across all dimensions, with median ratings of 4.00 from families and ECEs in most areas. No statistically significant differences were found between informant groups, suggesting consistency in perceived benefits. The use of satisfaction questionnaires to capture perspectives from key communication partners, although more established in the domain of pragmatics, is increasingly recognized as a valuable adjunct in broader language domains, including syntax (O'Shea et al., 2023; Pratt et al., 2022). This data offers an important perspective on the perceived relevance and impact of the intervention in naturalistic contexts.

Although percentile values were not included in the statistical analyses, they were examined descriptively to illustrate the clinical relevance of the intervention effects. The 16th percentile was adopted as a reference cutoff, in line with recent literature (e.g., Bao et al., 2024). At baseline, 90.30% (*n* = 28) of the total sample scored below this threshold on the SIN:TACS (85.70%, *n* = 12 in EG; 94.10%, *n* = 16 in CG) and 87.10% (*n* = 27) on the Subtest 3 of ALO (85.7%, *n* = 12 in EG; 88.20%, *n* = 15 in CG). After the intervention, all children scored above the 16th percentile on both measures. Additionally, 77.40% (*n* = 24) of the total sample reached or exceeded the 50th percentile on the SIN:TACS (71.40%, *n* = 10 in EG; 82.40%, *n* = 14 in CG) and 96.80% (*n* = 30) on the Subtest 3 of ALO (100%, *n* = 14 in EG; 94.10%, *n* = 16 in CG). These descriptive findings reinforce the clinical relevance of the observed results.

Despite the promising results, several methodological and contextual limitations must be acknowledged. The nonrandomized design and use of convenience sampling introduce potential selection bias and limit the internal validity of the findings. Although group equivalence at baseline was statistically verified, the lack of random allocation precludes causal inference. The relatively small sample size, although adequately powered for large effects, may not capture the full variability present in the broader population, and the absence of long-term follow-up restricts understanding of sustained effects.

Furthermore, the uneven sex distribution within the group could limit generalizability, especially considering that population-based studies have not consistently found sex differences in language outcomes among children with DLD (Norbury et al., 2016).

Another limitation concerns the age range within each condition group, which, although controlled for in statistical analyses, may have influenced responsiveness to intervention. Lastly, the choice of outcome measures, while standardized and validated for the target population, did not include structure-specific probes aligned directly with the syntactic targets addressed in the PROsyntax.

In light of these limitations, the present study provides preliminary evidence supporting the effects of PROsyntax for improving expressive and receptive skills in preschool-age children with DLD or ASD. It contributes to bridging a critical gap in early language intervention by demonstrating the program's feasibility and potential impact.

## Conclusion

This nonrandomized controlled trial evaluated the effects of the PROsyntax in 31 preschool-age children with syntactic impairments associated with DLD or ASD. Participants were allocated to experimental and control groups, and outcomes were assessed using validated standardized measures of expressive and receptive syntax.

Statistically significant improvements were observed in both linguistic components following the intervention, with robust effect sizes across conditions. These findings provide preliminary evidence that PROsyntax is an effective, structured, and clinically applicable intervention for addressing syntactic deficits in this population. The consistency of improvements across two sequentially implemented groups, along with positive feedback from families and ECEs, further supports the feasibility and relevance of the program in real-world settings. Nevertheless, additional studies with larger, randomized samples and longer-term follow-ups are required to strengthen the evidence base and explore potential differences in responsiveness between diagnostic conditions. Continued investigation will also contribute to refining intervention guidelines and informing tailored approaches in clinical practice.

## Supplemental Material

sj-docx-1-dli-10.1177_23969415251350586 - Supplemental material for The Effects of PROsyntax in Children with Developmental Language Disorder and Autism Spectrum Disorder: A Nonrandomized Controlled Trial Supplemental material, sj-docx-1-dli-10.1177_23969415251350586 for The Effects of PROsyntax in Children with Developmental Language Disorder and Autism Spectrum Disorder: A Nonrandomized Controlled Trial by Mafalda Azevedo, Alexandrina Martins, Tatiana Pereira, Pedro S. Couto and Marisa Lousada in Autism & Developmental Language Impairments

sj-docx-2-dli-10.1177_23969415251350586 - Supplemental material for The Effects of PROsyntax in Children with Developmental Language Disorder and Autism Spectrum Disorder: A Nonrandomized Controlled Trial Supplemental material, sj-docx-2-dli-10.1177_23969415251350586 for The Effects of PROsyntax in Children with Developmental Language Disorder and Autism Spectrum Disorder: A Nonrandomized Controlled Trial by Mafalda Azevedo, Alexandrina Martins, Tatiana Pereira, Pedro S. Couto and Marisa Lousada in Autism & Developmental Language Impairments
