# Supplementary material for: The Effects of PROsyntax in Children with Developmental Language Disorder and Autism Spectrum Disorder: A Nonrandomized Controlled Trial
Source: Autism Dev Lang Impair. 2025 Jun 17;10:23969415251350586. doi: 10.1177/23969415251350586 (PMC12181704; doi:10.1177/23969415251350586)
Supplement: sj-docx-2-dli-10.1177_23969415251350586 - Supplemental material for The Effects of PROsyntax in Children with Developmental Language Disorder and Autism Spectrum Disorder: A Nonrandomized Controlled Trial [file sj-docx-2-dli-10.1177_23969415251350586.docx]

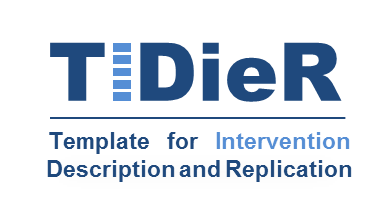
The TIDieR (Template for Intervention Description and Replication) Checklist*:

Information to include when describing an intervention and the location of the information

| **Item Item** | **Where located **** | |
| --- | --- | --- |
| **number** | Primary paper  (page or appendix number) | Other ^†^ (details) |
| **BRIEF NAME**  **1.** Provide the name or a phrase that describes the intervention. |  |  |
| **WHY** |  |  |
| **2.** Describe any rationale, theory, or goal of the elements essential to the intervention. | 1 (Title)  5-6 (Introduction) |  |
| **WHAT** |  |  |
| **3.** Materials: Describe any physical or informational materials used in the intervention, including those | 9 (Methods) |  |
| provided to participants or used in intervention delivery or in training of intervention providers. |  |  |
| Provide information on where the materials can be accessed (e.g. online appendix, URL). |  |  |
| **4.** Procedures: Describe each of the procedures, activities, and/or processes used in the intervention, | 9 (Methods) |  |
| including any enabling or support activities. |  |  |
| **WHO PROVIDED** |  |  |
| **5.** For each category of intervention provider (e.g. psychologist, nursing assistant), describe their | 9 (Methods) |  |
| expertise, background and any specific training given. |  |  |
| **HOW** |  |  |
| **6.** Describe the modes of delivery (e.g. face-to-face or by some other mechanism, such as internet or | 9 (Methods) |  |
| telephone) of the intervention and whether it was provided individually or in a group. |  |  |
| **WHERE** |  |  |
| **7.** Describe the type(s) of location(s) where the intervention occurred, including any necessary | 9 (Methods) |  |
| infrastructure or relevant features. |  |  |

| **WHEN and HOW MUCH**  9 (Methods)   1. Describe the number of times the intervention was delivered and over what period of time including   the number of sessions, their schedule, and their duration, intensity or dose.  **TAILORING**  9 (Methods)   1. If the intervention was planned to be personalised, titrated or adapted, then describe what, why,   when, and how.  **MODIFICATIONS**  9 (Methods)   1. **^ǂ^** If the intervention was modified during the course of the study, describe the changes (what, why,   when, and how).  **HOW WELL**  n/a   1. Planned: If intervention adherence or fidelity was assessed, describe how and by whom, and if any   strategies were used to maintain or improve fidelity, describe them.  n/a   1. **^ǂ^** Actual: If intervention adherence or fidelity was assessed, describe the extent to which the   intervention was delivered as planned. |  |
| --- | --- |

** **Authors** - use N/A if an item is not applicable for the intervention being described. **Reviewers** – use ‘?’ if information about the element is not reported/not sufficiently reported.

† If the information is not provided in the primary paper, give details of where this information is available. This may include locations such as a published protocol or other published papers (provide citation details) or a website (provide the URL).

ǂ If completing the TIDieR checklist for a protocol, these items are not relevant to the protocol and cannot be described until the study is complete.

- We strongly recommend using this checklist in conjunction with the TIDieR guide (see *BMJ* 2014;348:g1687) which contains an explanation and elaboration for each item.
- The focus of TIDieR is on reporting details of the intervention elements (and where relevant, comparison elements) of a study. Other elements and methodological features of studies are covered by other reporting statements and checklists and have not been duplicated as part of the TIDieR checklist. When a **randomised trial** is being reported, the TIDieR checklist should be used in conjunction with the CONSORT statement (see [www.consort-statement.org)](http://www.consort-statement.org/) as an extension of **Item 5 of the CONSORT 2010 Statement.**

When a **clinical trial protocol** is being reported, the TIDieR checklist should be used in conjunction with the SPIRIT statement as an extension of **Item 11 of the SPIRIT 2013 Statement** (see [www.spirit-statement.org](http://www.spirit-statement.org/)). For alternate study designs, TIDieR can be used in conjunction with the appropriate checklist for that study design (see [www.equator-network.org](http://www.equator-network.org/)).
